# HCV derived from sera of HCV-infected patients induces pro-fibrotic effects in human primary fibroblasts by activating GLI2

**DOI:** 10.1038/srep30649

**Published:** 2016-08-01

**Authors:** M. Granato, C. Zompetta, E. Vescarelli, C. Rizzello, A. Cardi, S. Valia, G. Antonelli, C. Marchese, M. R. Torrisi, A. Faggioni, M. Cirone

**Affiliations:** 1 Dept. of Experimental medicine, Sapienza University of Rome, Italy; 2 Dept. of Molecular medicine, Sapienza University of Rome, Italy; 3 Istituto Pasteur-Fondazione Cenci Bolognetti; Dept. Clinical and Molecular Medicine, Sapienza University of Rome, Italy; 4 Azienda Ospedaliera Sant’ Andrea, Rome, Italy

## Abstract

Hepatitis C virus (HCV) infection is a leading cause of liver fibrosis, especially in developing countries. The process is characterized by the excess accumulation of ECM that may lead, over time, to hepatic cirrhosis, liver failure and also to hepatocarcinoma. The direct role of HCV in promoting fibroblasts trans-differentiation into myofibroblasts, the major fibrogenic cells, has not been fully clarified. In this study, we found that HCV derived from HCV-infected patients infected and directly induced the trans-differentiation of human primary fibroblasts into myofibroblasts, promoting fibrogenesis. This effect correlated with the activation of GLI2, one of the targets of Hedgehog signaling pathway previously reported to be involved in myofibroblast generation. Moreover, GLI2 activation by HCV correlated with a reduction of autophagy in fibroblasts, that may further promoted fibrosis. GLI2 inhibition by Gant 61 counteracted the pro-fibrotic effects and autophagy inhibition mediated by HCV, suggesting that targeting HH/GLI2 pathway might represent a promising strategy to reduce the HCV-induced fibrosis.

Liver fibrosis is a response to chronic liver diseases of different etiologies, including infection by Hepatitis C virus (HCV). HCV is a RNA virus, with size of 50 to 80 nm in diameter, belonging to the Flaviviridae family, that exists in six different genotypes. It is an important cause of morbidity and mortality worldwide, since it has been reported that approximately 170 million of people are infected. HCV-driven liver fibrosis can progress to liver cirrhosis and hepatocarcinoma. Despite being a leading cause of morbidity and mortality, effective therapies able to reduce liver fibrosis are still lacking. Resident hepatic stellate cells (HSCs) and portal fibroblasts undergoing activation and trans-differentiation into myofibroblasts are the major fibrogenic cells^1^. The direct role of natural HCV in inducing pro-fibrotic effects in HSCs and fibroblasts has not been fully clarified. Because of the lack of an efficient cell culture system for viral growth and the difficulties to obtain natural HCV, the on-going studies are based on JFH1-HCV models. JFH1-HCV subgenomic replicon has been reported to infect embryonic fibroblasts^2^ and HSCs^3^. In addition, it has been recently shown that cell-cultured derived HCV obtained from the Huh7.5 cell line infected with the full-length genome of JFH-1 can infect liver myofibroblasts, stimulating their fibrogenic activities^4^.

The Hedgehog (HH) pathway regulates multiple processes involved in development and differentiation of tissues and organs during embryonic life^5^. In humans, three ligands, sonic hedgehog (SHH), desert hedgehog (DHH) and indian hedgehog (IHH), are responsible for HH canonical activation, by binding to the inhibitory receptor Patched (Ptch). As a consequence of their binding, the repression upon the transmembrane transducer Smoothened (Smo) is reduced and the signal is transduced to the downstream GLI transcription factors, namely GLI1, GLI2 and GLI3^5^. A non-canonical HH activation, independent of the presence of HH ligands, can also occur^6^. The inappropriate activation of HH pathway has been involved not only in several cancers^7^,^8^ but also in organ fibrosis^9^. Moreover, it has been reported that transgenic mice expressing SHH in the liver developed liver cirrhosis and were more susceptible to carcinogenesis induced by other oncogenes^10^. In particular, it has been reported that GLI2, one of the transcription factors activated downstream of HH signaling pathway, induced hepatic stellate cells (HSCs) to acquire myofibroblastic phenotype^11^ and seems to be the more important GLI effector in the induction of renal fibrosis^12^. HH pathway may also regulate autophagy^13^ and in particular GLI2 activation negatively influences the autophagic process^14^.

Autophagy is a catabolic process involved in tissue development and differentiation that also serves to maintain normal tissue homeostasis^15^. Its dysfunction is involved in the pathophysiology of many human disorders, including fibrosis. Indeed, autophagy induction may reduce liver fibrosis^16^ and inhibit HSC proliferation and extracellular matrix deposition in mouse models^17^. Moreover, autophagy induction reduces type I collagen accumulation in HSCs and promotes its degradation, reducing kidney fibrosis^18^. However, regarding the survival and activation of HSCs, the role of autophagy remains still controversial^19^,^20^. Interestingly, HH pathway has been reported to be up-regulated by HCV and to promote HCV replication in hepatocytes^21^.

In this study we investigated whether natural HCV, obtained from sera of HCV-infected patients, as previously reported^22^, would infect and directly promote a pro-fibrotic activation in human primary fibroblasts. We also asked whether natural HCV would activate GLI2 in human fibroblasts and modulate autophagy in these cells. The understanding of the molecular mechanism/s underlying HCV-induced fibrosis is of fundamental importance in the attempt to develop new therapeutic strategies to counteract this pathological process, alongside with the discovering of more effective therapies aimed at reducing the viral load of HCV^23^.

## Results

### HCV derived from sera of HCV-infected patients infects and induces pro-fibrotic effects in human primary fibroblasts

**Figure 1:**
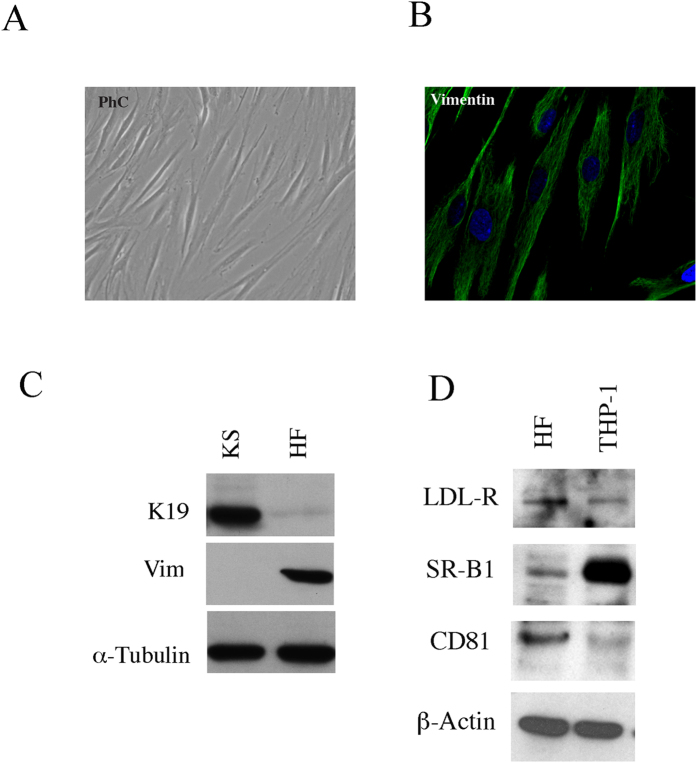
Characterization of human primary skin fibroblasts and HCV receptor expression. Human fibroblasts, isolated from skin of healthy donors were analysed (**A**) by phase contrast optical microscopy to assess their morphology (**B**) by IFA to detect vimentin expression and (**C**) by western blot analysis to detect the expression of vimentin (mesenchymal marker) and the lack of K19 (epithelial marker). (**D**) The expression of the putative HCV receptors CD81, SR-B1 and LDLr was investigated by western blotting using THP-1 cells as control.

**Figure 2:**
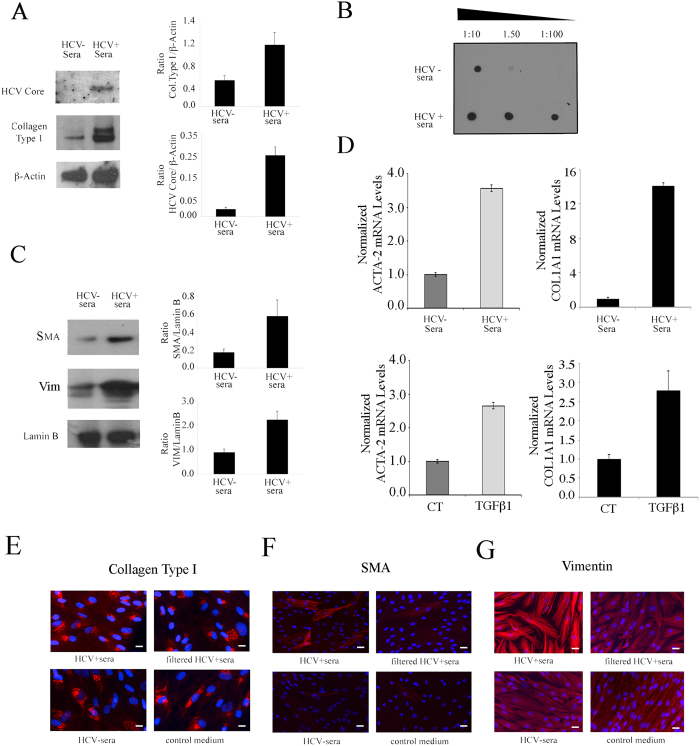
HCV induces pro-fibrotic effects in human primary fibroblasts. Human fibroblasts exposed to HCV-containing sera or to HCV-negative sera (20% of the final volume, for four days) and (**A**) HCV core protein and Type I collagen expression was investigated by western blot analysis or (**B**) collagen release was investigated by dot blot analysis. (**C**) SMA and vimentin expression was analysed in fibroblasts exposed to HCV-positive sera or negative sera. Lamin B or β-Actin were used as loading controls. A representative experiment out of 3 is shown. The histograms represent the mean plus SD of the densitometric analysis of the specific molecules on loading control of 3 different experiments. (**D**) SMA (ACTA-2) and collagen type I (COL1A1) RNA expression as evaluated by qRT-PCR. mRNA levels were normalized to GAPDH mRNA expression. Error bars represent standard deviations. For this experiment, fibroblasts were exposed to HCV-positive and HCV-negative sera or as positive control, they were treated for TGFβ1 (10 ng/ml, for four days). Mean plus SD of three indepent experiments is shown. Fibroblasts exposed to HCV-positive sera or to the same sera from which viral particles removed through ultrafiltration or HCV-negative sera or control medium were analysed for the expression of (**E**) type I collagen (**F**) SMA and (**G)** vimentin, by IFA. Blue DAPI staining is also shown. A representative experiment out of 3 is shown. Bar: 5 micron.

Human primary fibroblasts, isolated from skin of healthy donors, were characterized based on their morphology (Fig. 1A), assessment of vimentin (mesenchimal marker) expression by IFA (Fig. 1B) and by western blot together with the lack of K19 (epithelial marker) expression (Fig. 1C). The expression of the putative HCV receptors by these cells was then assessed. We first analysed whether they expressed CD81 that is considered to be one of the most important among the putative HCV receptors^24^,^25^. By western blot analysis, we found that this molecule was expressed by primary fibroblasts (Fig. 1D). Then the expression of SR-B1, another receptor molecule known to be essential for HCV infection together with CD81 (Kapadia S D 2007 j virol) was explored and, as shown in Fig. 1D, SR-B1 was expressed by these cells. Interestingly, we found that fibroblasts expressed also LDL receptor (Fig. 1D) which is particularly important, since it is used by HCV for the *in-vivo* infection (Molina S. *et al.* 2007) given that the virus travels bound to the LDL into the the plasma (Burlone M E 2009). As control for the expression of all above reported HCV putative receptors THP1 cells were used. The western blot results were also confirmed by IFA (data not shown). Next, we investigated whether natural HCV, derived from HCV-infected patients with high levels HCV RNA IU/ml of circulating HCVRNA, (N2 × 10^6^), would directly infect and induce pro-fibrotic effects in human primary fibroblasts. At this aim, these cells were exposed to sera from HCV-infected patients or to HCV-negative patients, as control (20% of the final volume). After 4 days of *in-vitro* culture, the expression of HCV core protein was investigated by western blot analysis. The results shown in Fig. 2A indicate that fibroblasts exposed to HCV-containing sera were HCV-infected, expressing HCV core protein and concomitantly showed a higher expression level of type I collagen, the fibrillar collagen mainly produced in the course of fibrosis. Type I collagen production were also evaluated by dot blot analysis and accordingly, a higher amount of collagen was found to be released in the supernatants of HCV-exposed fibroblasts in comparison with the controls (Fig. 2B). Then, the expression of several markers of myofibroblast trans-differentiation such as smooth muscle actin and vimentin was evaluated by western blot analysis in fibrobasts exposed to HCV-positive or negative sera. The results obtained indicate that fibroblasts exposed to HCV-containing sera displayed a higher expression of SMA and vimentin (VIM) (Fig. 2C) in comparison to fibroblasts exposed to HCV-negative sera. The increased expression of SMA and type I collagen was then confirmed at RNA level by qRT-PCR (Fig. 2D). As control, we treated fibroblasts with TGF beta1 (TGFβ1) (10 ng/ml) and found that SMA and collagen type I expression increased in TGFβ1-treated cells in comparison with the untreated control (Fig. 2D). Finally, the induction of the fibrotic effects by HCV was investigated by immunofluorescence assay (IFA) and, to confirm that HCV itself was responsible for the induction of the pro-fibrotic phenotype, fibroblasts were also exposed to the same HCV-positive sera from which the viral particles were removed through ultrafiltration (HCV filtered). A regular culture medium was included as control in addition to HCV-negative control sera. We found that fibroblasts exposed to HCV-containing sera showed higher expression of type I collagen (Fig. 2E), SMA (Fig. 2F) and vimentin (Fig. 2G) in comparison to fibroblasts exposed to filtered HCV sera or HCV-negative sera or control medium, indicating that the natural HCV contained in the sera of infected patients was able to infect and directly induce trans-differentiation of human primary fibroblasts into myofibroblasts, promoting fibrosis.

### HCV derived from sera of HCV-infected patients activates GLI2 in human primary fibroblasts

**Figure 3:**
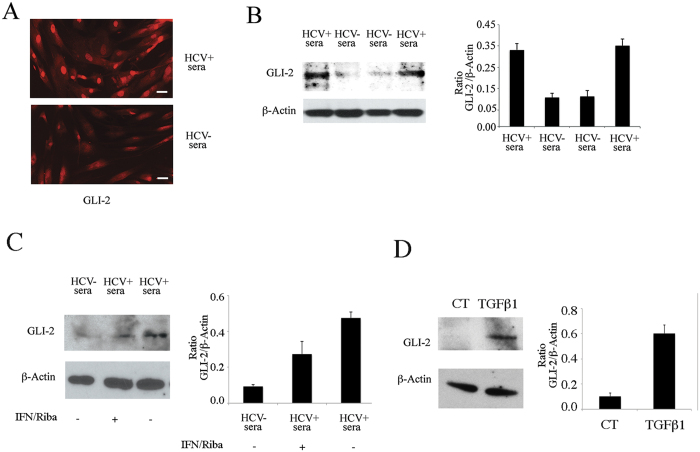
HCV activates GLI2 in human primary fibroblasts. (**A**) GLI2 expression was evaluated in fibroblasts exposed to HCV-containing sera or to HCV-negative sera by IFA, Bar: 5 micron or (**B**) by western blot analysis. (**C**) GLI2 expression was assessed in fibroblasts exposed to HCV-containing sera in the presence or in the absence of pegylated interferon and ribavirin or (**D**) treated with TGF beta 1 (TGFβ1). β-Actin was used as loading control. A representative experiment out of 3 is shown. The histograms represent the mean plus SD of the densitometric analysis of the ratio of GLI2 on loading control of 3 different experiments.

Searching for the molecular pathways underlying the pro-fibrotic activation of fibroblasts, we found that GLI2, a target of HH pathway^5^ was activated and localized mainly in the nuclei of HCV-exposed fibroblasts, as evaluated by IFA (Fig. 3A). Then, a western blot analysis performed with a cellular lysate enriched of the nuclear fraction, showed a higher GLI2 expression in fibroblasts exposed to HCV-containing sera in comparison to the fibroblasts exposed to HCV negative sera (Fig. 3B). To assess the direct role of HCV infection in the activation of this pathway, we added pegylated interferon alfa plus Ribavirin concomitantly to the HCV-containing sera. These drug combination has been reported to be effective in HCV anti-viral therapy^26^. A reduction of GLI2 expression was observed in the presence of these antiviral agents (Fig. 3C), indicating that HCV infection plays an important role in the mediating this effect. As control, we treated fibroblasts with TGF beta1 and found that, similarly to HCV, it was able to activate GLI2 (Fig. 3D), as previously reported^27^. This finding suggests that HCV could induce the production of TGFβ1 by primary fibroblasts, as it does in other cell types^28^ and that this could contribute to GLI2 activation together with the effects mediated by the virus itself. GLI2 pathway has been strongly linked to fibrogenesis^12^, and the results obtained in this study suggest that it could play a role in the fibrotic process induced by HCV in fibroblasts.

### HCV derived from sera of HCV-infected patients reduces autophagy in human primary fibroblasts

**Figure 4:**
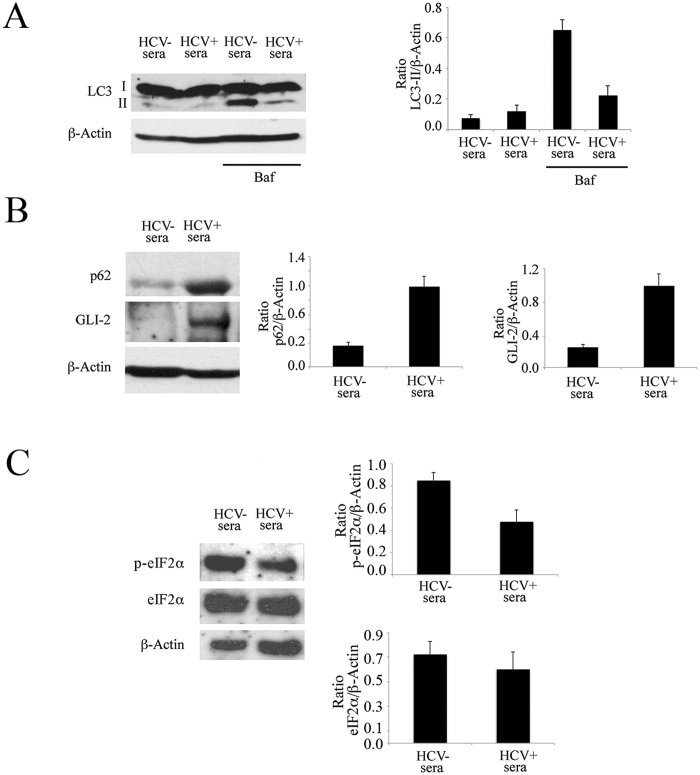
HCV reduces autophagy in human primary fibroblasts. (**A**) Expression of LC3-I/II in the presence or in the absence of Bafilomycin A1 (baf). (**B**) of p62 and GLI2 and (**C**) of phospho- and total eIf2α in fibroblasts exposed to HCV-containing sera or HCV-negative sera. β-Actin was used as loading controls. A representative experiment out of 3 is shown. The histograms represent the mean plus SD of the densitometric analysis of the ratio of the specific molecules on loading control of 3 different experiments.

GLI2 activation has been reported to negatively regulate autophagy^14^, thus we then asked whether GLI2 activation by HCV would result in autophagy reduction in human fibroblasts. To this aim, the autophagic marker LC3-I/II was analysed by western blot immunoblotting. Since the lipidated form of LC3 (LC3-II) is formed and degraded through the autophagic process, we used bafilomycin A1 (baf), which blocks autophagosome-lysosome fusion, to evaluate LC3II formation. We found that LC3II expression was reduced in fibroblasts exposed to HCV-containing sera in the presence of baf, in comparison to the same cells exposed to HCV-negative control sera (Fig. 4A), suggesting that HCV reduces autophagy in fibroblasts. The reduction of autophagy was further confirmed by the increase of p62 (Fig. 4B), a protein mainly degraded through autophagy that indeed accumulated in fibroblasts exposed to HCV-containing sera, concomitantly with GLI2 activation (Fig. 4B). We then found that GLI2 activation correlated with the reduction of the phosphorylated form of eiF2alfa (Fig. 4C), which is essential for autophagy induction^29^. These results suggest that reduction of eiF2alfa phosphorylation might link HCV-mediated GLI2 activation with autophagy inhibition. Since autophagy has been shown to influence the fibrotic process by reducing collagen degradation^18^, it is possible that autophagy inhibition by HCV-mediated GLI2 activation could contribute to the pro-fibrotic effects in human primary fibroblasts.

### Gant 61 counteracted the pro-fibrotic effects and autophagy inhibition mediated by HCV

**Figure 5:**
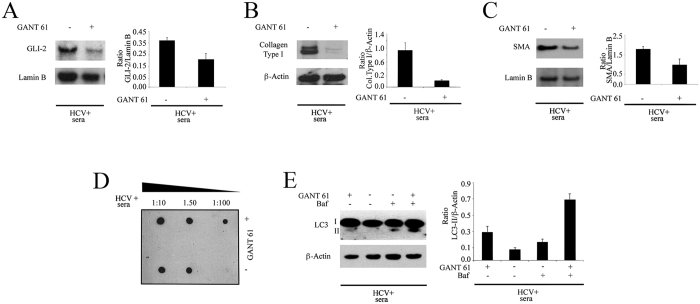
Gant 61 counteracts the pro-fibrotic effects and autophagy inhibition induced by HCV. (**A**) GLI2, (**B)** type I collagen and (**C**) SMA expression in HCV-exposed fibroblasts pre-treated or not with GANT61 61 (5 μM). Lamin B or β-Actin were used as loading controls. (**D**) Dot-blot analysis showing type I collagen release in the culture conditioned medium of fibroblasts pre-treated or not with Gant 61 and exposed to HCV-containing sera. (**E**) Expression of LC3-I/II in the presence or in the absence of bafilomycin in fibroblasts pre-treated or not with Gant 61 and exposed to HCV-containing sera. β-Actin was used as loading control and a representative experiment out of 3 is shown. The histograms represent the mean plus SD of the densitometric analysis of the ratio of specific molecules on loading control of 3 different experiments.

Next, we investigated whether GLI2 activation would be responsible for the pro-fibrotic effects and autophagy inhibition mediated by HCV in primary fibroblasts. At this purpose, fibroblasts were pre-treated with Gant 61 (5 μM), a small molecule able to inhibit GLI2^30^, before exposure to HCV-containing sera. Gant 61 pre-treatment, reduced GLI2 activation (Fig. 5A) and resulted in a decrease of type I collagen (Fig. 5B) and SMA (Fig. 5C) expression in cells exposed to HCV. Moreover, collagen release was reduced by Gant 61, as assessed by dot blot analysis (Fig. 5D). Finally, the impact of Gant 61 on the autophagic process was investigated. As shown in Fig. 5E, LC3-II increased in HCV-infected fibroblasts pre-treated with Gant 61 in the presence of baf, in comparison to the same HCV-infected untreated fibroblasts, indicating that this small molecule was able to restore autophagy in HCV-infected fibroblasts. All together these results suggest that GLI2 activation could be one of the mechanisms underlying the fibrogenesis induced by HCV in human primary fibroblasts and that targeting GLI2 by Gant 61 could be a useful therapeutic strategy to counteract autophagy inhibition and the pro-fibrotic effects induced by HCV.

## Discussion

Liver fibrosis as well as fibrosis of other organs is a process characterized by the excess accumulation of extracellular matrix (ECM) that is often related to a process of chronic inflammation^31^. HSCs and portal fibroblasts, differentiating into myofibroblasts, are the most important cells involved in the liver fibrosis. HSCs are the main fibrogenic cell type in pericentral areas while portal fibroblasts may predominate when liver injury occurs around portal tracts. Inflammatory cells that also cross-talk with fibroblasts, also participate to fibrogenesis^32^. HCV chronic infection, autoimmune and biliary diseases and alcoholic steatohepatitis are the leading causes of liver fibrosis, process that often progress to hepatic cirrhosis and also to hepatocarcinoma^28^,^33^. HCV infection of hepatocytes increases production of ROS and transforming growth factor β1 (TGFβ1) that, in turn, stimulate HSCs and portal fibroblasts to become myofibrobasts^34^. The pathogenesis of HCV-induced liver fibrosis and whether the HCV specific proteins play a role in promoting fibrosis is poorly understood, due to the to lack of an efficient cell culture system for viral growth and the lack of models of persistent HCV infection. In this study, we used natural HCV, derived from sera of HCV-infected patients, to infect human primary fibroblasts and investigate the outcome of HCV infection on fibrogenesis. We observed that HCV directly promoted primary fibroblast trans-differentiation into myofibroblats, based on the increased expression of SMA, vimentin and collagen expression/production. We found that these effects were mediated by the virus itself, since its removal through ultrafiltration completely abrogates the pro-fibrotic activity of HCV-positive sera. Several HCV proteins, including core, have been previously reported to stimulate inflammation and fibrosis in HSCs^35^. We found that fibroblasts exposed to HCV-containing sera expressed HCV core antigen, suggesting that this molecule could be involved in the induction of the pro-fibrotic phenotype. Searching for molecular mechanisms underlying myofibroblast trans-differentiation, we found that GLI2 was activated in fibroblasts exposed to HCV-containing sera in comparison to HCV negative sera. Interestingly, in this study we observed a reduction of GLI2 activation in the presence of pegylated interferon plus Ribaviran anti-viral agents, which confirms that HCV itself plays a direct role in maintaining its activation. GLI2 is one of the targets activated downstream of HH pathway signaling that has been previously linked to organ fibrosis of several organs such as kidney^12^ and liver^36^,^37^. Its importance has been strengthened by the results obtained by the inhibition of GLI2 that led to a reduction of myofibroblast generation and of kidney fibrosis^38^,^39^. Besides liver fibrosis, the activation of HH pathway has been also linked to hepatocarcinoma and its inhibition has been shown to be able to revert both pathological processes in a mouse model^40^. HH pathway plays also a role in the epithelial-mesenchymal transition (EMT) that also promotes fibrosis, thus it is emerging as a promising target for the treatment of fibrosis-related diseases^9^. Of note, HH inhibition has been shown to reduce fibrosis also in the course of Systemic Sclerosis (SSc)^41^, whose etiopathology has been recently linked to Epstein-Barr virus infection^42^. The results obtained in this study indicate that Gant 61 reduced the pro-fibrotic effects mediated by HCV in primary fibroblasts in terms of SMA and collagen expression and production. Interestingly, we found that Gant 61, concomitantly to the reduction of fibrotic markers, also counteracted autophagy inhibition induced by HCV. Accordingly, GLI2 activation has been previously reported to negatively regulate autophagy^13^,^14^ and autophagy inhibition to promote the fibrotic process^16^. As a possible mechanism leading to autophagy inhibition, we have shown that HCV reduced the phosphorylation of eIF2α, a mechanism used also by other viruses to interfere with the autophagic process^29^. It would be interesting, in future studies, to explore whether autophagy would be inhibited in fibroblasts by other viruses such as Human Hepatitis B (HBV), whose infection has been also strongly linked to liver fibrosis^43^. Autophagy is often manipulated by viruses according to their convenience^22^,^44^,^45^,^46^ and HBV, similarly to HCV may induce autophagy to promote its replication^47^. However, autophagy seems to be reduced in HBV-associated tumorigenesis as well as in the tumor tissues of HBV-associated hepatocellular carcinomas^48^,^49^. Interestingly, HBV can also activate HH pathway to promote carcinogenesis^50^. Another virus that promotes the fibrotic process and is involved in the pathogenesis of SSc is EBV^42^ and it will be also important to explore whether GLI2 and autophagy manipulation would be involved in mediating the pro-fibrotic effects. In conclusion, the results obtained in this study unveil for the first time a link between fibrosis induced by HCV in primary fibroblasts with GLI2 activation and autophagy inhibition. More importantly, we found that the treatment with GANT61 was able to counteract all these effects, encouraging the use of this small molecule in the reduction of HCV-induced fibrosis. Its use is even more promising when considering that HH pathway inhibition has been shown also to be able to reduce HCV replication^21^ that strongly correlated with the outcome of HCV-related diseases.

## Materials and Methods

### Human Sera

Human sera were obtained from healthy donors (HCV, HIV and HBsAg-negative) or from HCV-infected patients (HIV and HBsAg-negative), in which the viral load was measured by VERSANT HCV RNA 3.0 Assay (Siemens Health Care, Australia). The limits of detection (LOD) and quantification (LOQ) were 15 HCV RNA IU/ml according to the assay package inserts. Valid results were reported quantitatively (IU/ml), as “positive b LOQ” (if the value obtained was under the LOQ), or as “target not detectable”.

Sera from 3 patients, which were anti-IgG HCV positive, as measured by ELISA (ADVIA Centaur, Siemens Health Care, Australia) and who had high levels HCV RNA IU/ml of circulating HCVRNA, (N2 × 10^6^), genotype 1, were used throughout the study.

In some experiments, the HCV-positive sera were ultrafiltered with 30 k Vivaspin columns (Sartorius, Goettingen, Germany, Europe). This ultrafiltration process, validated for removal of a variety of enveloped and non-enveloped viruses with size from 70 nm to 30 nm, was used to remove HCV particles.

### Cell culture and reagents

Primary cultures of human fibroblasts (HF) were established from 1 cm^2^ skin biopsy from healthy donor. Dermis was excised from the biopsy sample and subjected to enzymatic dissociation for 20 min at 37 °C. Then cells were seeded onto collagen IV (10 mg/mL) coated culture plates and maintained in Dulbecco’s modified Eagle medium (DMEM) (Sigma, St. Louis, MO, USA; D6546), supplemented with 10% Fetal Bovine Serum (FBS) (Euroclone, Milan, Italy; ECLS0180L), L-glutamine (100 μg/ml) (Aurogene, Rome, Italy; AU-X0550-100) and streptomycin/penicillin (100 U/ml) (Aurogene, Rome, Italy; AU-L0022-100) in 5% CO2 at 37 °C, with medium change twice a week.

To better characterize HF cell cultures, we examined their morphology by phase contrast microscopy. Furthermore, to better clarify HF mesenchymal origin, we performed immunofluorescence analysis to evaluate vimentin expression. The expression of a specific mesenchymal marker (vimentin) and the lack of expression of an epithelial marker (K19), were also assessed by western blot analysis, confirming HFs mesenchymal origin. Fibroblasts characterization is shown in Fig. 1A–C.

Human Fibroblasts were grown in culture-dishes (∅60 mm) (SPL Lifescience Inc., Korea) in 3 ml or cultured on coverslips (∅12 mm) (Carl Roth GmbH + Ko.KG, Karlsruhe, Germany) in 1 ml of DMEM.

THP-1 cells were purchased from ATCC (USA).

To investigate autophagy, in some experiments, fibroblasts were treated with Bafilomycin A1 (20 nM) (Santa Cruz Biotechnology Inc., Europe), an inhibitor of vacuolar-H^+^-ATPase, for the last three hours.

As HCV antiviral agent, a combination of Pegylated Interferon alfa (100 U/ml) and Ribavirin (50 ug/ml), both purchased from Roche (Italy), was used.

TGFβ1, purchased from Sigma Aldrich (Italy) was used at 10 ng/ml to treat fibroblasts, for 4 days.

GANT 61 (5 μM) (Enzo Life Science, Lausen, Switzerland) was used as chemical inhibitor of GLI proteins, as previously described^51^.

### HCV infection and treatments of Human Fibroblasts

HCV infection was performed by exposing HF to HCV-positive or HCV-positive filtered sera or HCV-negative for 60 minutes at 37 °C. Cells were then cultured for 4 days in a medium containing 20% of the same sera or control medium in 1.5 ml (dishes) or 0.5 ml (coverslips).

In same experiments Pegylated Interferon alfa and Ribavirin (used at 100 U/ml and 50 ug/ml, respectively) were added concomitantly to HCV-positive sera.

To inhibit the Hedgehog pathway, HF were pre-treated with GANT61 (5 μM) for 30 min and then exposed to the HCV-positive sera.

### Antibodies

In this work we used the following primary antibodies:

Mouse monoclonal anti-Vimentin (1:100) (Dako, Glostrup, Denmark), mouse monoclonal anti-Smooth Muscle Actin^15^ (1:100) (Dako, Glostrup, Denmark), rabbit polyclonal anti-Collagen Type 1 (1:1500) (Novus Biologicals, Cambridge, UK), mouse monoclonal anti-K19 (Santa Cruz, Biotechnology Inc., Europe), rabbit polyclonal anti-GLI2 (1:100) (Santa Cruz Biotechnology Inc., Europe), rabbit polyclonal anti-peIF2α (1:200) (Cell Signaling, Europe) and rabbit polyclonal anti-eIF2α (1:500) (Cell Signaling, Europe). Anti CD81, anti-SR-B1 and anti-LDL recetor were purchased by Novus Biologicals, Cambridge, UK and used 1:100. The HCV infection was controlled using a rabbit polyclonal anti-Hepatitis C Virus Core Antigen (HCV Core) (1:100) (Abcam, Cambridge, UK) antibody. To study autophagy, we used the following primary antibodies: rabbit polyclonal anti-LC3 (1:1000) (Novus Biologicals, Cambridge, UK), mouse monoclonal anti-p62 (1:1000) (BD Transduction Laboratories, San Jose, CA, USA).

Monoclonal mouse anti-α-Tubulin (1:1000), (Sigma Aldrich, Europe), anti-β-Actin (1:10000) (Sigma Aldrich, Europe), and goat polyclonal anti-Lamin B (1:100) (Santa Cruz Biotechnology Inc., Europe) were used as loading controls. The monoclonal anti-mouse IgG-HRP (Santa Cruz Biotechnology Inc., Europe), the polyclonal anti-rabbit IgG-HRP (Santa Cruz Biotechnology Inc., Europe) and the polyclonal anti-goat IgG-HRP (Santa Cruz Biotechnology Inc., Europe) were used as secondary antibodies in western blot analysis. All the primary and secondary antibodies were diluted in PBS-0.1% Tween20 solution containing 3% of BSA.

### Western blot analyses

1.5 × 10^6^ HF were washed twice with 1X PBS solution and centrifuged at 1500 rpm for 5 minutes at 4 °C. The cells were lysed in a 1X RIPA buffer containing 150 mM NaCl, 1% NP-40, 50 mM Tris-HCl (pH8), 0.5% deoxycholic acid, 0.1% SDS, protease and phosphatase inhibitors. Then, the lysates were incubated on ice for 20 min and centrifuged at 14000 rpm for 20 min at 4 °C. The first supernatant (labeled as I) was separated from the remaining pellet (including cellular debris and also the insoluble fraction containing the nuclear matrix). Subsequently, 50 μl of 1X RIPA plus 0.1% Triton X-100, was added to the pellet that it, kept on ice, was sonicated for 45 seconds at the power of 180 watts (in round of 10 seconds). Then, the samples were centrifuged as described before and the second supernatant (labeled as II) were mixed to the first one (I). 30 μg of protein lysates were subjected to electrophoresis on 4–12% NuPage Bis-Tris (Life Technologies, Carlsbad, CA, USA) or 3–8% (GLI2 protein) NuPage Tris-Acetate gels (Life Technologies, Carlsbad, CA, USA), according to the manufacturer’s instruction. To evaluate the LC3-I/II, the cell lysates were denatured in 3X Loading Protein Buffer (150 mM Tris (pH 6.8), 6% SDS, 30% Glycerol, 30 mM EDTA, 0.2% Bromophenol Blue) for 5 min at 100 °C and run on 15% gel (30% acrylamide/Bis Solution 29:1) (Biorad, Hercules, CA, USA) in Tris-Glycine-SDS buffer. Then, the gels were transferred to Nitrocellulose Membranes (Biorad, Hercules, CA, USA) for 2 hrs in Tris-Glycine buffer. The membranes were blocked in PBS 0.1% Tween20 solution containing 3% of BSA, probed with specific primary and secondary antibodies. Finally, bands on membranes were revealed using an ECL Blotting Substrate (Advansta, Menlo Park, CA, USA).

### Dot Blot

Supernatants recovered from HF cultured in DMEM medium in the presence of HCV-negative and HCV-positive with or without GANT61 (5 μM) (Enzo Life Science, Lausen, Switzerland) were used to perform a dot blot assay to detect the release of Collagen Type I. Briefly, the supernatants were diluted in 1X PBS at different concentrations (1:10; 1:50; 1:100). Then, 3 μl of samples were spotted on a rectangular nitrocellulose membrane and allowed to dry for some minutes. The membranes were blocked in PBS 0.1% Tween20 solution containing 5% of BSA for 1 hour and then were incubated with a rabbit polyclonal anti-Collagen Type 1 (1:1500) (Novus Biologicals, Cambridge, UK) antibody for 1 hour at room temperature. An anti-rabbit IgG-HRP (Santa Cruz Biotechnology Inc., Europe) was used as secondary antibody for 30 min at room temperature. All the primary and secondary antibodies were diluted in PBS-0.1% Tween20 solution containing 3% of BSA. Finally, a chemiluminescence kit, ECL Blotting Substrate (Advansta, Menlo Park, CA, USA), was used to detect signals.

### Quantitative Real-Time PCR

Cells were processed for total RNA extraction with the use of TRIzol reagent (Invitrogen, Karlsruhe, Germany). cDNA was generated with oligo (dT) from 1 μg of RNA using the SuperScript III Reverse Transcriptase Kit (Invitrogen). For ACTA-2 and Collagen-1 mRNA detection, commercially available specific probes were used (Applied Biosystems by Life Technologies, Carlsbad, CA, USA) (COL1A1,4331182; Applied Biosystems by Life Technologies, Carlsbad, CA, USA) (ACTA2, 4331182; Applied Biosystems by Life Technologies, Carlsbad, CA, USA). A total of 2 μl/well of template was added to the sample wells along with Taqman Universal PCR master mix at a concentration of 1x and water to a volume of 25 μl/well. Assays were performed in triplicate on an ABI 7500 Real-Time instrument (Applied Biosystems) using the following conditions: 50 °C for 2 minutes, 95 °C for 10 minutes, and then 95 °C for 15 seconds and 60 °C for 1 minute, repeated 40 times. Relative quantification was performed using GAPDH mRNA as an endogenous control: for each examined sample, ACTA-2 and Collagen-1 mRNA expression data were normalized to the GAPDH expression.

### Indirect Immunofluorescence assay (IFA)

The human fibroblasts were seeded on coverslips in 24-wells plates, exposed or not exposed to HCV-positive filtered or not filtered or negative sera and grown in DMEM medium containing the same sera (20% of the final volume) for 4 days before immunofluorescence (IFA). In same experiments, fibroblasts were pre-treated with GANT61 (5 μM) before exposing to the HCV-positive sera. IFA was performed as described previously^52^. Briefly, HF-cultured on coverslips were washed three times in 1X PBS and then fixed in 2% paraformaldehyde diluted in 1X PBS for 30 min at 25 °C. The cells were permeabilized with a 0.1% Triton X-100 solution for 5 min. After several washing with 1X PBS, HF were incubated with the following primary antibodies: mouse monoclonal anti-Vimentin (1:100 in 1X PBS) (Dako, Glostrup, Denmark), mouse monoclonal anti-Smooth Muscle Actin (1:100 in 1X PBS) (Dako, Glostrup, Denmark) and rabbit polyclonal anti-GLI-2 (1:100 in 1X PBS) (Santa Cruz Biotechnology Inc., Europe). Subsequently, they were washed three times in 1X PBS and incubated with polyclonal Cy3-conjugated or FITC-conjugated sheep-anti mouse (1:1000 in 1X PBS) or Rhodamine (TRITC) Goat anti-Rabbit (1:300 in 1X PBS) (Jackson Immunoresearch Laboratories, West Grove, PA, USA) for 30 minutes at room temperature. Nuclei were stained with 4′,6′-diamino-2-phenylindole (DAPI) for 1 min at room temperature. Finally, cells were resuspended in a 1X PBS-glycerol solution (1:1) and the immunofluorescence was analyzed by using an Apotome Axio Observer Z1 inverted microscope, equipped with an AxioCam MRM Rev.3 at 40× magnification.

### Densitometric Analysis

The quantification of proteins bands was performed by densitometric analysis using the Image J software, which it was downloaded from NIH web site (http://imagej.nih.gov).

### Ethics statement

The study was approved by the ethical Committee of “Policlinico Umberto I”.

The experiments were carried out in accordance with the approved guidelines and the “informed” consent was obtained from all subjects.

## Additional Information

**How to cite this article**: Granato, M. *et al.* HCV derived from sera of HCV-infected patients induces pro-fibrotic effects in human primary fibroblasts by activating GLI2. *Sci. Rep.* **6**, 30649; doi: 10.1038/srep30649 (2016).
